# Allyl isothiocyanate (AITC) activates nonselective cation currents in human cardiac fibroblasts: possible involvement of TRPA1

**DOI:** 10.1016/j.heliyon.2020.e05816

**Published:** 2020-12-31

**Authors:** Gaku Oguri, Toshiaki Nakajima, Hironobu Kikuchi, Shotaro Obi, Fumitaka Nakamura, Issei Komuro

**Affiliations:** a Department of Cardiovascular Medicine, University of Tokyo, Tokyo 113-8655, Japan; b Department of Cardiovascular Medicine, Dokkyo Medical University, Tochigi, 321-0293, Japan; c Teikyo University Chiba Medical Center, Ichihara, 299-0111, Chiba, Japan

**Keywords:** Human cardiac fibroblast, Transient receptor potential ankyrin 1, TRPA1, Allyl isothiocyanate, Methylglyoxal, Nonselective cation channels

## Abstract

The effects of allyl isothiocyanate (AITC), transient receptor potential ankyrin 1 (TRPA1) agonist, on cultured human cardiac fibroblasts were examined by measuring intracellular Ca^2+^ concentration [Ca^2+^]_i_ and whole-cell voltage clamp techniques. AITC (200 μM) increased Ca^2+^ entry in the presence of [Ca^2+^]_i_. Ruthenium red (RR) (30 μM), and La^3+^ (0.5 mM), a general cation channel blocker, inhibited AITC-induced Ca^2+^ entry. Under the patch pipette filled with Cs^+^- and EGTA-solution, AITC induced the current of a reversal potential (Er) of approximately +0 mV. When extracellular Na^+^ ion was changed by NMDG^+^, the inward current activated by AITC was markedly reduced. La^3+^ and RR inhibited the AITC-induced current. The conventional RT-PCR analysis, Western blot, and immunocytochemical studies showed TRPA1 mRNA and protein expression. The present study shows the first evidence for functional Ca^2+^-permeable nonselective cation currents induced by AITC, possibly via TRPA1 in human cardiac fibroblast.

## 1 Introduction

Transient receptor potential ankyrin 1 (TRPA1) channel is a nonselective cation channel having ankyrin repeats on the N-terminus [1]. It is predominately expressed in sensory neurons [2], but there have been several reports that TRPA1 channel is identified in non-neuronal cells including lung fibroblasts [3] and arterial endothelial cells (ECs) [4]. And, TRPA1 channel plays an important role on cardiovascular diseases such as heart failure and myocardial fibrosis [5]. Some papers also illustrated that TRPA1 channel was identified in cardiomyocytes and activation of TRPA1 dose-dependently enhanced the contractile function and intracellular Ca^2+^ concentration ([Ca^2+^]_i_), but not in cardiomyocytes prepared from *Trpa1*^−/−^ mice [6, 7, 8]. Wang *et al.* [9, 10] have shown that TRPA1 expression was increased in mouse and human failure hearts, and the channel inhibition improved cardiac hypertrophy and function in the mouse pressure overload model due to transverse aortic constriction (TAC). Conklin et al. [8] also reported that the TRPA1 expression in cardiomyocytes was high in the intercalated disks and involved in acrolein-induced Ca^2+^ increase. According to these studies, TRPA1 appears to play a role in regulating cardiac function under various conditions including heart failure [5, 11].

Cardiac fibroblasts activated by various stimuli, such as myocardial injury and heart failure, mainly produce extracellular matrix (ECM), which play an essential role in cell signaling and fibrotic responses [12, 13, 14]. A number of evidences indicate that Ca^2+^-dependent pathways are involved in fibroblasts proliferation, differentiation and ECM production [15]. Among them, transient receptor potential (TRP) protein superfamily is composed of cation channels in various cells such as cardiac fibroblasts [16]. They play an integral role in pathophysiological and physiological conditions. Hatano *et al.* [17] showed the vanilloid type 4 transient receptor potential channel (TRPV4) in rat cardiac fibroblasts, by using 4 alpha-phorbol 12,13-didecanoate, a TRPV4 agonist. In recent years, we have reported that TRPA1 is identified in human cardiac fibroblasts (hCFs), and TRPA1 channel blocker inhibited Ca^2+^ influx by methylglycoxal (MG), then inhibiting the proliferation [18]. Okada *et al.* [19] also reported that the TRPA1 channel inhibitor reduced fibrosis and inflammation by inhibiting transforming growth factor-β1 signaling cascades in ocular fibroblasts. Thus, these studies have shown that TRPA1 channel may become a potential therapeutic target for fibrotic reaction. However, Ca^2+^-dependent pathways in hCFs are still not clearly known.

Therefore, the present study is to clarify whether TRPA1 channels can function in hCFs, by using the [Ca^2+^]_i_ measurements and the patch-clamp techniques.

## 2 Methods

### 2.1 Cell culture of hCFs

Human adult ventricular cardiac fibroblasts (ACBRI 5118) were commercially purchased from DS PHARMA BIOMEDICAL Co., Ltd (Osaka, Japan) [20]. The cells were cultured in Cell System Corporation with defined cell boost (CSC Catalog 4ZO-50) and 10% serum (37 °C under 5% CO_2_). The cells at confluence were detached using 0.25% trypsin and cultured into the medium. The medium was changed twice a week. Cells at passage 3–6 were stripped from the culture dish with 0.25% trypsin and used for the experiments.

### 2.2 Drugs

The composition of control Tyrode solution was as follows (in mM): NaCl 136.5, KCl 5.4, CaCl_2_ 1.8, MgCl_2_ 0.53, glucose 5.5, and N-2-hydroxyethylpiperazine-N′-ethane sulfonic acid (HEPES)-NaOH buffer 5.5 (pH 7.4). The Ca^2+^-free bath solution contained EGTA (0.5 mmol/L) in the Ca^2+^-free Tyrode solution. In N-methyl-d-glucamine^+^ (NMDG^+^) solutions, extracellular Na^+^ was changed with equimolar concentration of N-methyl-d-glucamine^+^ (NMDG^+^). The patch pipette was filled with the following Cs^+^-internal solution (in mM); CsCl 140, EGTA 5, MgCl_2_ 2, Na_2_ATP 3, GTP 0.1, and HEPES-CsOH buffer 5 (pH 7.2). Allyl isothiocyanate (AITC), a selective TRPA1 agonist [21, 22], was purchased from Wako Pure Chemical Industries (Osaka, Japan). MG and ruthenium red (RR), a non-selective membrane-impermeable cation channel inhibitor including TRPA1 [1, 22, 23, 24, 25], were obtained from Nakarai Tesque (Kyoto, Japan). HC030031 (2-(1,3-Dimethyl-2,6-dioxo-1,2,3,6-tetrahydro-7H-purin-7-yl) N-(4-isopropylphenyl) acetamide), a selective TRPA1 blocker [26], was obtained from Abcam Biochemicals (Cambridge, UK). Fura-2 acetoxymethyl ester (fura-2/AM, molecular probes) and lanthanum (La^3+^) were obtained from Dojin Chemicals.

### 2.3 Measurement of [Ca^2+^] _i_

[Ca^2+^] _i_ was measured with the fluorescence method as previously reported [27, 28]. The fibroblasts were trypsinized, washed, and adjusted to a cell density of 10^6^ cells/ml and loaded with fura-2 AM (2 μM) (37 °C under 5% CO_2_). Then the fura-2AM containing medium was removed, and fluorescent cells in suspensions were measured while stirred in a cuvette placed by a spectrofluorometer (CAF-100; Jasco, Tokyo, Japan). The excitation wavelengths were 340 and 380 nm, and the emission was 500 nm. The fluorescence intensity ratio of F340/F380 was used as an indicator of [Ca^2+^]_i_.

### 2.4 Recording techniques

Membrane currents were recorded under the whole-cell voltage clamp techniques with a patch-clamp amplifier (EPC-7, List Electronics, Darmstadt, Germany) [29, 30]. The patch electrode had a tip resistance of 3–5 MΩ. All data were acquired, and analyzed on Power Macintosh 7100/80 by using the PULSE + PULSEFIT software (HEKA Electronic) and Igor PRO (Wave Metrics, Lake Oswego, OR).

### 2.5 Immunofluoresence staining

The immunofluoresence staining was performed on hCFs with anti-TRPA1 (extracellular) antibody (ACC-037, Alomone Labs, Jerusalem, Israel). The hCFs were cultured on collagen I-coated chamber slide (177402, Nunc, Rochester, NY), fixed with 2% paraformaldehyde in phosphate-buffer saline (PBS). The cells were blocked with PBS containing 1% bovine serum albumin. They were incubated for overnight with primary antibody in the presence or absence of blocking peptide (BLP-CC037, Alomone Labs) diluted with 1% bovine serum albumin in PBS into 1:400. Alexa Fluor 555 labeled Donkey anti-rabbit IgG antibody (A31572, ThermoFisher SCIENTIFIC, Waltham, MA) diluted 1:1000 was used to visualize the channel expression. The hCFs were mounted by using Fluoroshield with DAPI (ImmunoBioScience Corp, Mukilteo, WA) to visualize nuclei. A fluorescence microscope system (BZ-X700, KEYENCE Corp, Osaka, JAPAN) was used for observations.

### 2.6 Western blotting

The hCFs were washed in PBS, and lysed in RIPA buffer with 1% Nonidet P-40, 0.1% sodium dodecylsulfate, 0.5% sodium deoxycholate, and 1% protease inhibitor cocktail (25955, Nacalai Tesque, Kyoto, Japan). Samples were centrifuged at 14,000 rpm (4 °C, 25 min), and total proteins in the supernatant were separated. The ProteoExtract™ Native Membrane Protein Extraction Kit (Merck KGaA, Darmstadt, Germany) was used to isolate native membrane protein. The isolated proteins were kept at -80 °C before sodium dodecylsulfate-polyacrylamide gel electrophoresis (SDS-PAGE).

Proteins were separated on a 7% polyacrylamide gel at 250 V and transferred onto a PVDF membrane (Amersham Hypond-P, Cytiva, Tokyo, Japan) at 130 mA with semi-dry method for 60 min. After that, the membrane was blocked with Blocking One (Nacarai Tesque, Kyoto, Japan) for 1 h at room temperature. It was then exposed to anti-TRPA1 antibody (NB110-40763, Novus Biologicals, Littleton, CO, USA) diluted to 1:500 with Signal Enhancer HIKARI Solution A (Nacalai Tesque, Kyoto, Japan) at 4 °C overnight. Subsequently, the probed membrane was washed three times by using 0.1% tween20 in Tris-buffered saline (TBS-T) and incubated with anti-rabbit IgG linked to peroxidase (7074s, Cell Signaling Technology, Danvers, MA, USA) diluted to 1:2000 with Signal Enhancer HIKARI Solution B (Nacalai Tesque, Kyoto, Japan). After washes, the bound antibodies were revealed via Chemi-Lumi One Super (Nacalai Tesque, Kyoto, Japan) and detected by a LuminoGraph I (ATTO, Tokyo, Japan).

### 2.7 RNA extraction, and reverse transcription-PCR (RT-PCR)

All cellular RNAs were extracted from hCFs using ISOGEN II (NIPPON GENE CO. LTD. Tokyo, Japan). For RT-PCR, complementary DNA (cDNA) was synthesized from 40ng of total RNA for 10μl of each reverse transcription reaction with a ReverTra Dash (TOYOBO CO., LTD. Osaka, Japan). The reaction mixture was subjected to PCR amplification with specific forward and reverse oligonucleotide primers for 40 cycles with heat denaturation, annealing, and extension. The PCR products were size-fractionated on 2% agarose gels and controlled under the blue LED light. The primers were determined based on the sequence of human TRPA1. The forward primer sequence was 5′- TGGTGCACAAATAGACCCAGT-3′ and the reverse primer sequence was 5′-TGGGCACCTTTAGAGAGTAGC-3′.

### 2.8 Data analysis

All values are expressed as means ± S.D.

## 3 Results

### 3.1 TRPA1 mRNA and protein in hCFs

**Figure 1:**
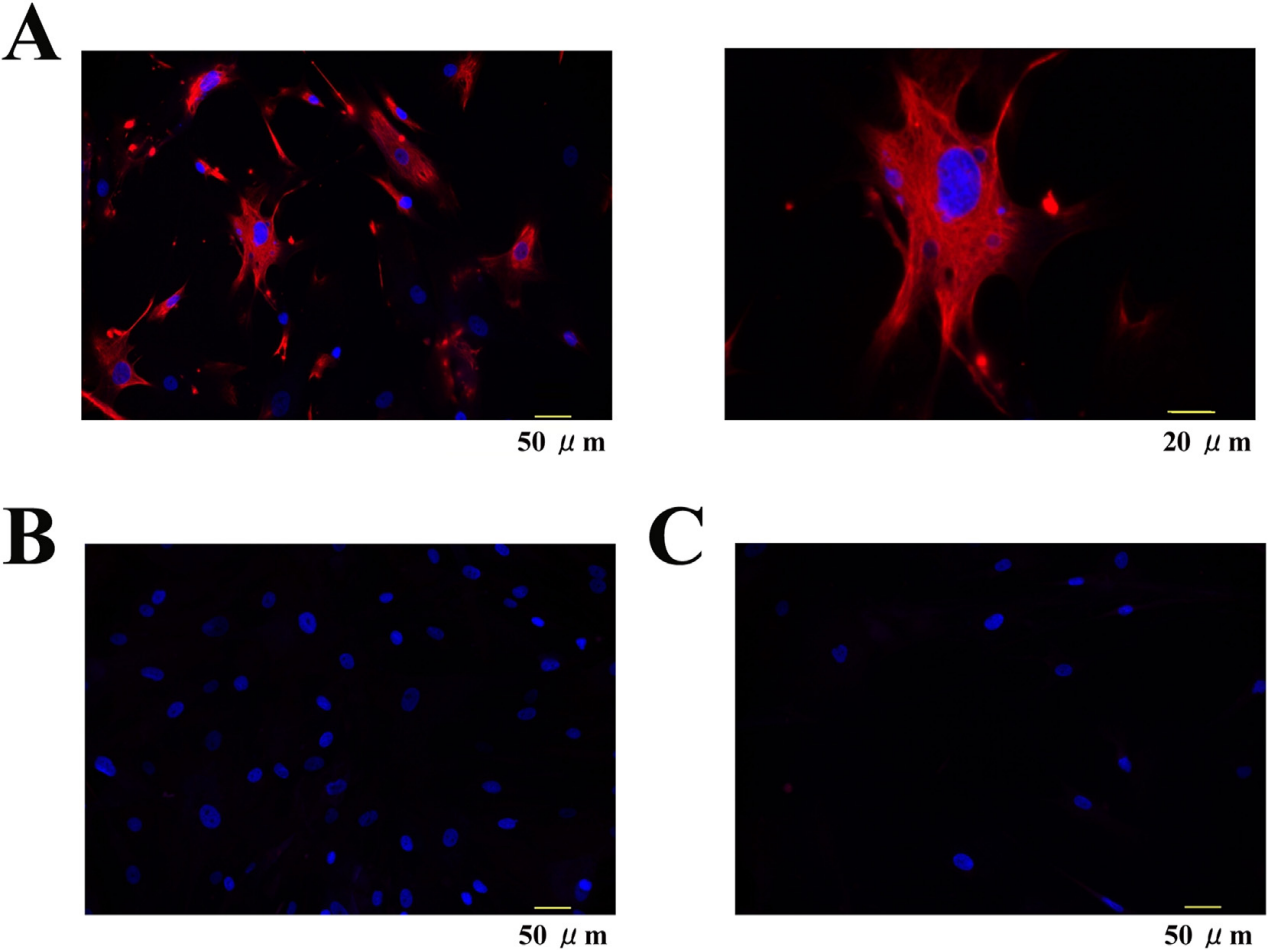
TRPA1 expression in human cardiac fibroblasts (hCFs). Immunostaining for TRPA1 in A. The right picture indicates large scale of left picture. In B, the cells were co-incubated with a blocking peptide. Note that co-incubation with a blocking peptide decreased the staining. Negative control in the absence of the antibody in C. Double staining of nuclei DAPI to visualize nuclei is also shown. The scale is shown in lower part of each picture.

The expression of TRPA1 channel was confirmed by immunocytochemistry (Figure 1A). The hCFs were counterstained with DAPI to visualize nuclei, and double staining of nucleus and TRPA1 protein. TRPA1 staining was blocked by coincubation of TRPA1 antibody with a blocking peptide, which decreased TRPA1 staining (Figure 1B). The channel expression was not observed in negative controls with normal rabbit IgG, when primary antibody was absent (Figure 1C).

**Figure 2:**
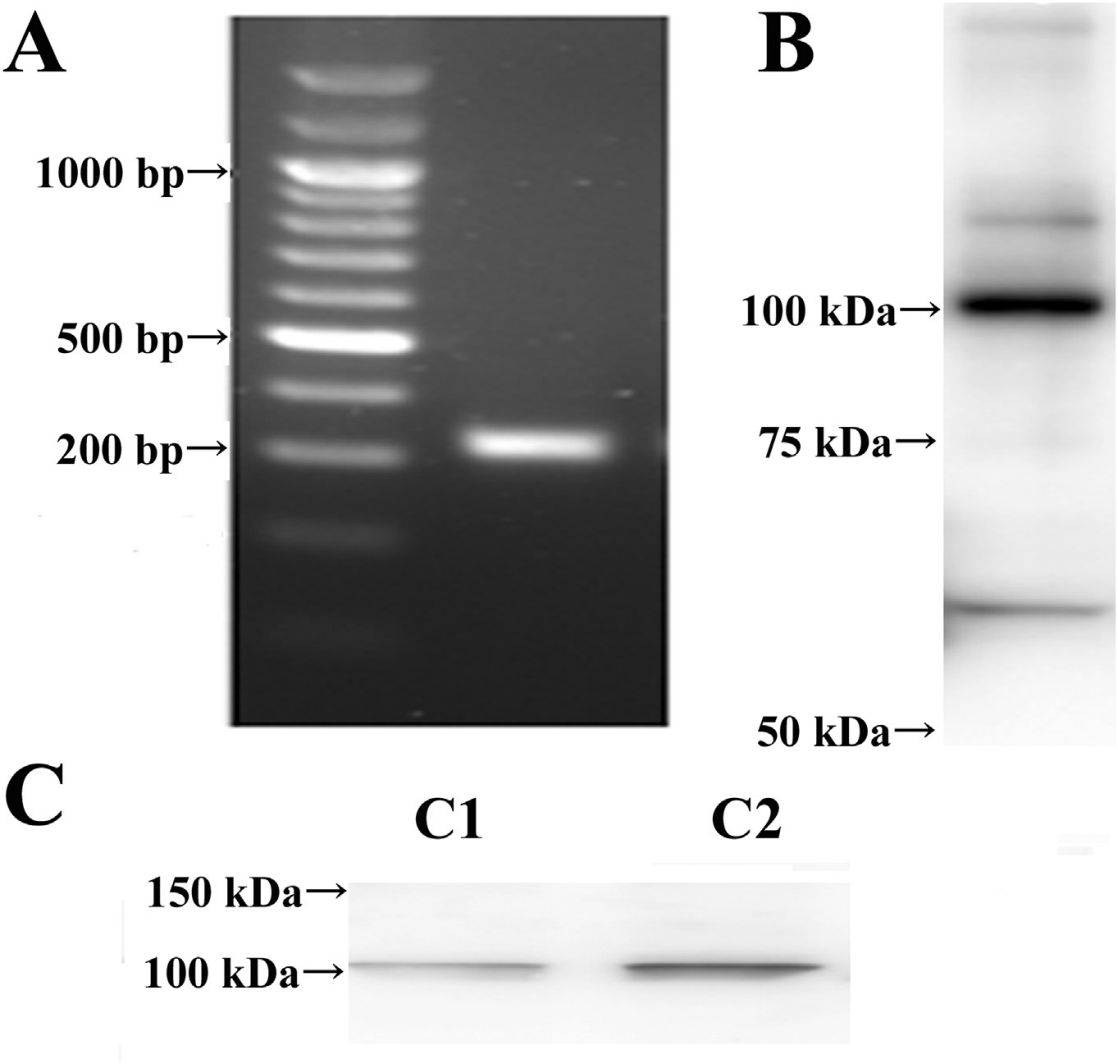
TRPA1 mRNA and protein expression. A: Expression of α subunit gene of TRPA1 channel mRNA in hCFs. Left, marker. B: Western blotting of TRPA1 protein isolated from total cells. C: Western blotting of TRPA1 protein isolated from membrane fraction. Two representative data (C1 and C2) are shown.

Next, we examined TRPA1 mRNA expression in hCFs (Figure 2A). TRPA1 mRNA was detected in hCFs. The amplitude of cDNA fragments was of predicted molecular size (317 bp), identical to cDNA fragments amplified from reverse transcribed mRNA (Figure 2A). In addition, to identify the channel protein expression, Western blot analysis of total cell protein was performed. A specific antibody for TRPA1 channel protein showed a strong band at approximately 100 kDa (Figure 2B). Similarly, Western blot analysis of membrane protein showed a band (Figure 2C). These immunocytochemistry and western blotting studies revealed the TRPA1 protein expression in hCFs.

### 3.2 Effects of allyl isothiocyanate (AITC) on [Ca^2+^]_i_

**Figure 3:**
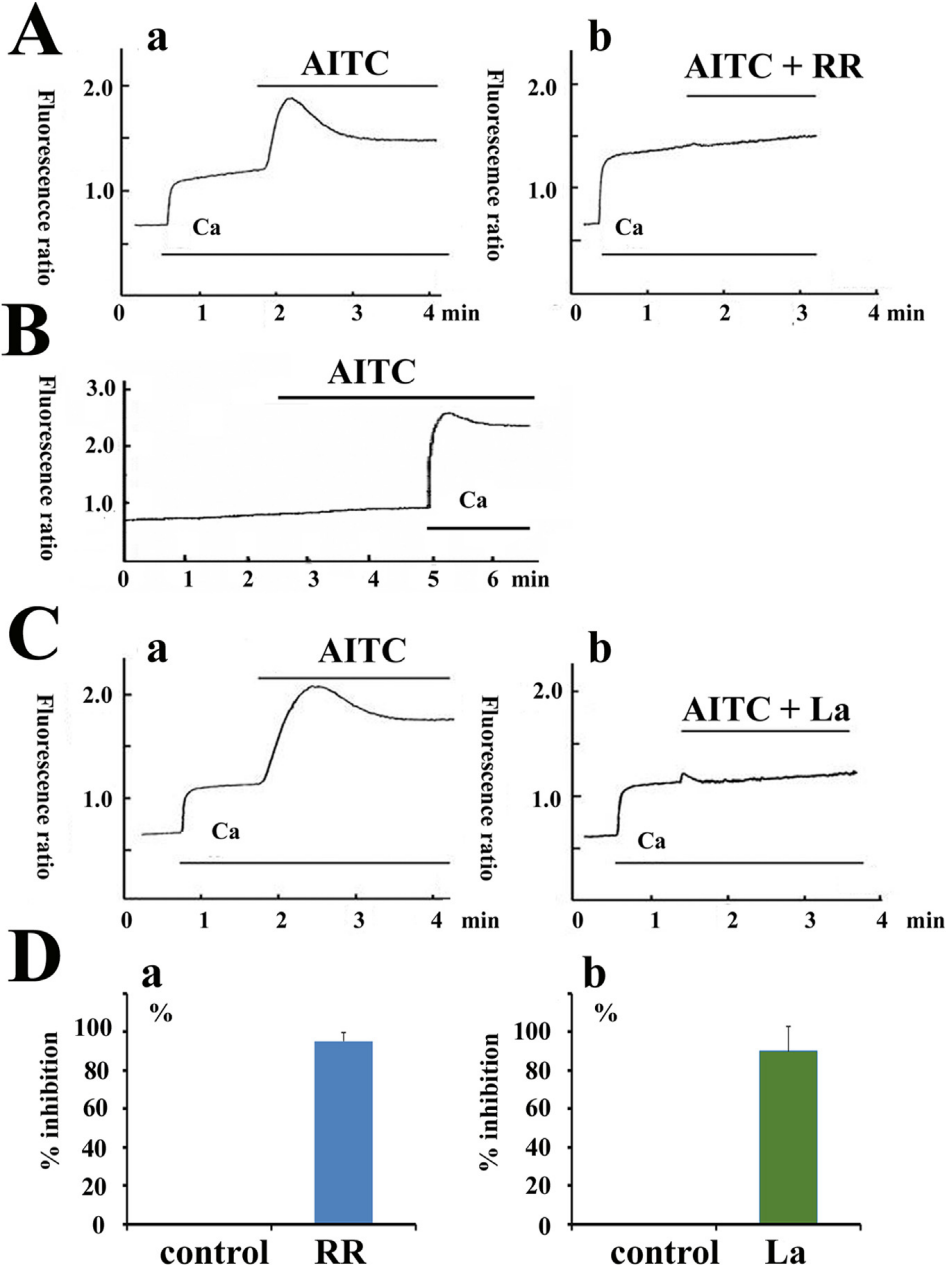
Effects of allyl isothiocyanate (AITC) on intracellular Ca^2+^ concentration [Ca^2+^]_**i**_ A: Effects of AITC and ruthenium red (RR) on [Ca^2+^]_i**.**_ AITC (200 μM; Aa) increased [Ca^2+^]_i._. However, the addition of RR (30 μM; Ab) with AITC reduced the AITC-induced [Ca^2+^]_i**,**_ compared with AITC only (Aa). B: Effects of AITC on [Ca^2+^]_i._ in the absence of extracellular Ca^2+^. C: Effects of La^3+^ (0.5 mM) on [Ca^2+^]_i._ The additional application of La^3+^ with AITC (Bb) abolished the AITC-induced [Ca^2+^]_i,_ compared with AITC only (Ba). D: Inhibitory effects of RR (30 μM; Da) and La^3+^ (0.5 mM; Db) on AITC-induced [Ca^2+^]_i._ increase. The increased value in F340/F380 induced by AITC was obtained in the bath solution with and without drugs. The increased value in the absence of drugs is considered as 100%. The percent inhibition of these agents is illustrated in D.

To examine whether TRPA1 can function in hCFs, [Ca^2+^]_i_ was measured. Figure 3 shows the effects of AITC on [Ca^2+^]_i_. In the presence of extracellular Ca^2+^, the application of AITC (200 μM) induced a rapid increase of [Ca^2+^]_i_ and subsequently decreased to a steady-state level (Figs. 3Aa & Ca). In contrast, AITC did not affect [Ca^2+^]_i_ in the absence of extracellular Ca^2+^ (Figure 3B). Furthermore, we examined the effects of various blockers on AITC-induced [Ca^2+^]_i_ rise. First, the effects of RR, a non-selective TRP blocker including TRPA1 [1, 22, 23, 24, 25], were investigated (Figure 3A). The additional of RR (30 μM, Figure 3Ab) with AITC markedly inhibited AITC-induced [Ca^2+^]_i_, compared with control cells (Figure 3Aa). RR (30 μM) inhibited it by 95 ± 4% (Figure 3Da. n = 8). La^3+^ (0.5 mM), an agent known to block various subtypes of TRP channels [30, 31], also inhibited AITC-induced [Ca^2+^]_i_ rise (Figure 3Cb), compared with control cells (Figure 3Ca). La^3+^ inhibited it by 90 ± 12% (Figure 3Db, n = 5).

### 3.3 AITC-induced nonselective cation currents in hCFs

**Figure 4:**
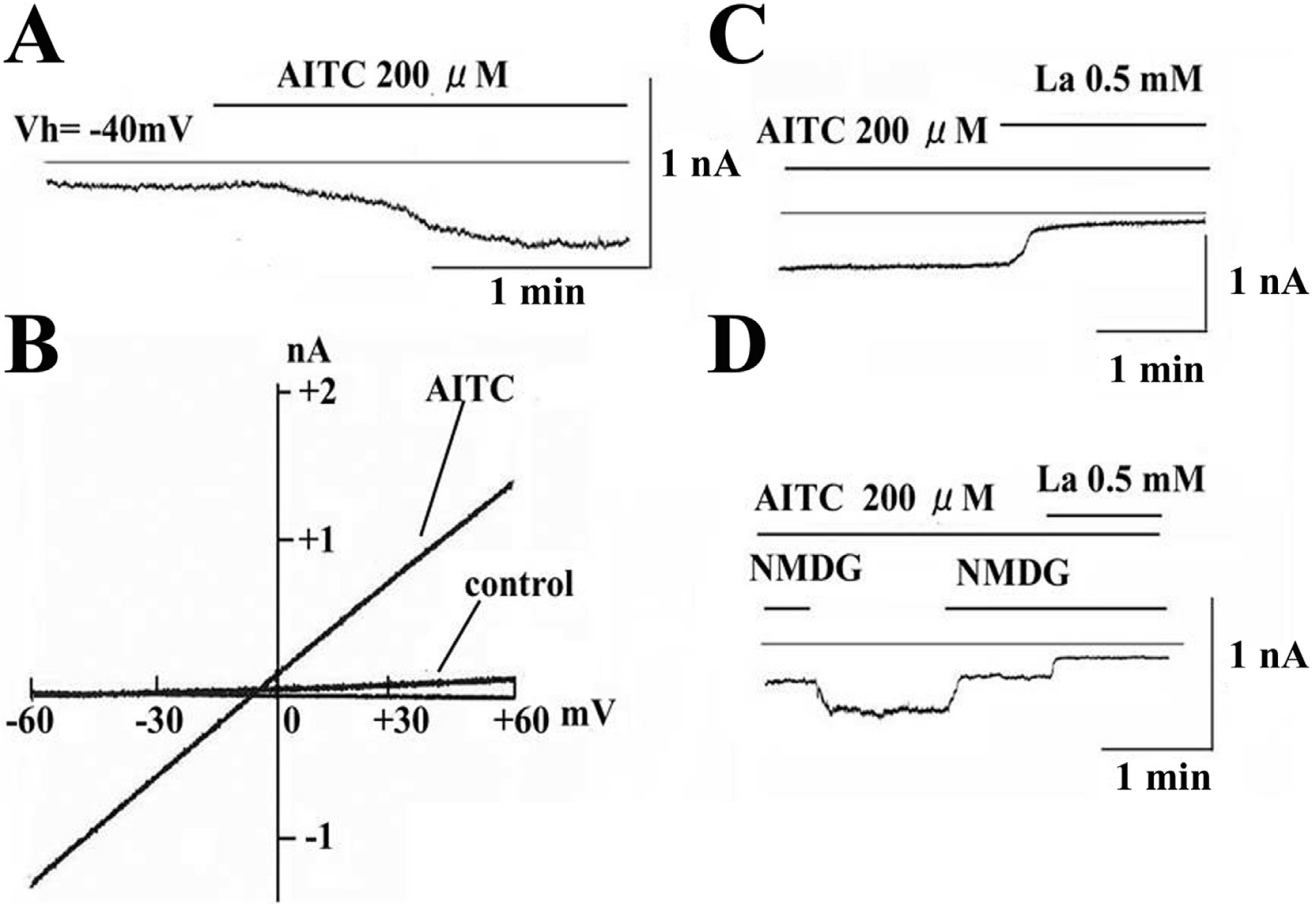
Allyl isothiocyanate (AITC) activates nonselective cation currents A: Effects of allyl isothiocyanate (AITC) on membrane currents. B: The current-voltage relationships obtained by applying ramp pulses from -60 to +60 mV (200 ms duration) in control and in the presence of AITC (200 μM), C: Effects of La^3+^ (0.5 mM) on AITC-induced current. D: Effects of total replacement of extracellular Na^+^ to NMDG^+^, an impermeable cation, on the AITC-induced current.

**Figure 5:**
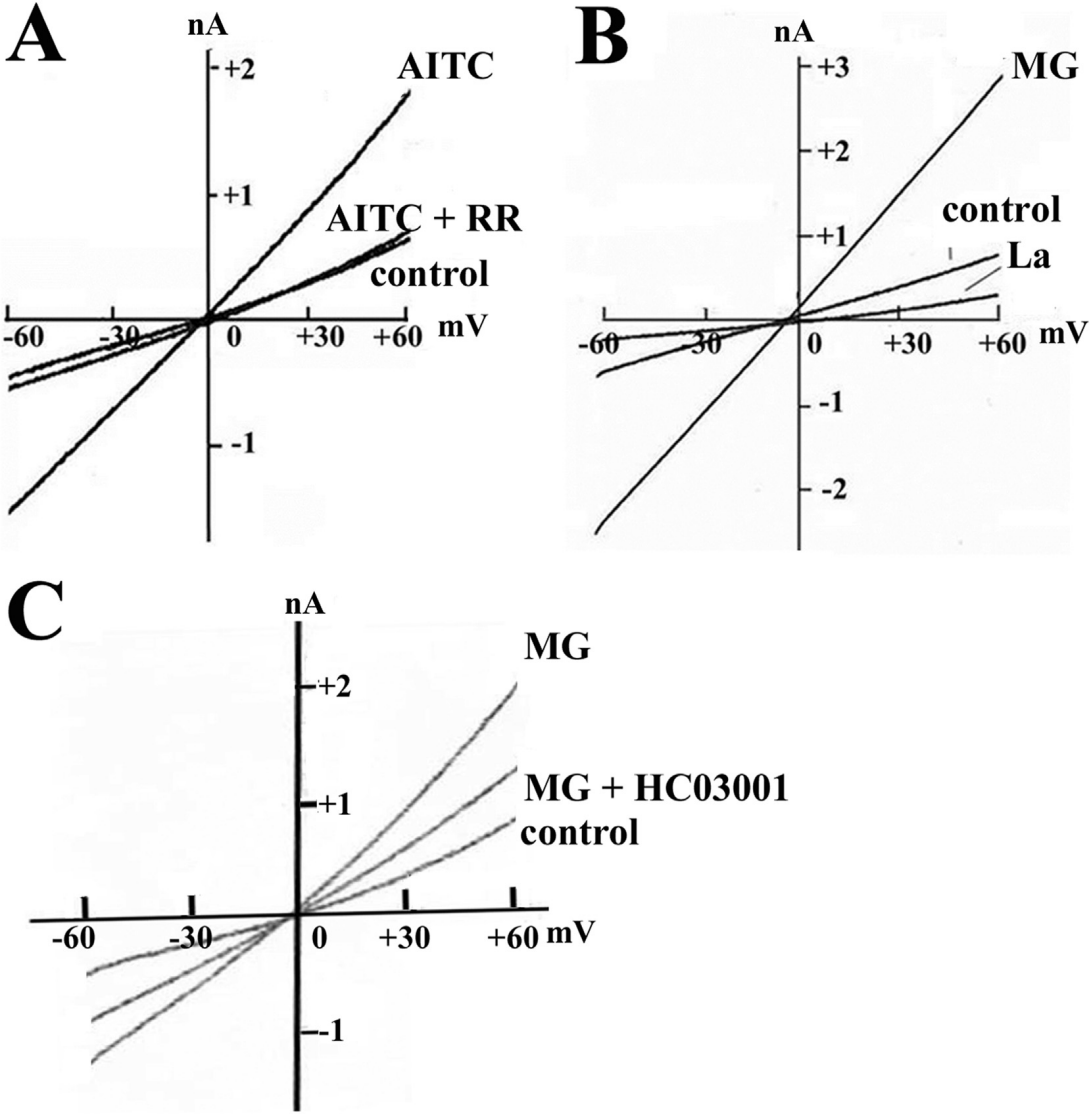
Activation of nonselective cation currents by AITC, and methyglyoxal (MG), and antagonistic effects of ruthenium red (RR) and HC030031. A: Effects of RR on AITC-induced currents. The I–V relationships obtained by ramp pulses were shown in control, in the presence of AITC (200 μM), and AITC plus RR (30 μM). B: The current-voltage (I–V) relationships of MG-induced current. The I–V relationships were shown in control, in the presence of MG (100μM), and MG plus La^3+^ (0.5 mM). C: Effects of HC030031 (10 μM) on MG-induced current. The I–V relationships were shown in control, in the presence of MG (100 μM), and MG plus HC030031 (10 μM).

We examined the effects of AITC (200 μM) on membrane currents in hCFs. The cells were held at -40 mV. The patch pipette was filled with the Cs^+^-internal solution. AITC (200 μM) increased the inward current at a holding potential of -40 mV (Figure 4A). The current-voltage (I–V) relationships were obtained by ramp pulses from -60 to +60 mV (200 ms duration). The I–V relationships were shown in control, and in the presence of AITC (200 μM). The I–V relationships of the AITC-induced current was obtained by subtracting the control current from the current in the presence of AITC. It was almost linear with the reversal potential (Er) of -3 ± 2 mV (n = 5). La^3+^ markedly inhibited the AITC-induced current (Figure 4C). Furthermore, replacement of extracellular Na^+^ to NMDG^+^, an impermeable cation, decreased the AITC-induced current (Figure 4D). Effects of RR (30 μM) on AITC-induced currents were investigated by using ramp pulses (Figure 5A). The I–V relationships are shown in control, in the presence of AITC (200 μM), and AITC plus RR (30 μM). RR markedly inhibited the AITC-induced currents at any potential with Er of approximately +0 mV. However, RR did not significantly affect the control (background) I–V relationships in the absence of AITC (data not shown).

We have previously showed that MG increased [Ca^2+^]_i_ in hCFs [17]. Figure 5B shows the I–V relationships of MG-induced currents. Similarly, in cases with AITC (Figure 5A), MG (100 μM) induced nonselective cation currents with Er of approximately +0 mV (Figure 5B). La^3+^ (0.5 mM) completely abolished the MG-induced currents. However, La^3+^ further decreased the background current with Er of approximately +0 mV. HC030031 (10 μM) significantly decreased MG-induced nonselective cation currents (Figure 5C).

## 4 Discussion

The major findings of the present study are as follows. 1) AITC increased [Ca^2+^]_I_ and activated nonselective cation currents with Er of approximately +0 mV. 2) La^3+^and RR, a general nonselective TRP channel blocker, inhibited the AITC-induced current and calcium responses. 3) Existence of TRPA1 channel mRNA and proteins were shown by the RT-PCR analysis, immunocytochemistry and western blotting studies. These results provide the evidence for functional existence of nonselective cation channels, possibly via TRPA1 channels, activated by AITC in hCFs, by using patch clamp techniques.

TRPA1 was first isolated by a screen of transformation-sensitive protein in cultured fibroblasts [32]. We have recently showed the evidence for TRPA1 channel mRNA, and protein expression in hCFs, and MG increases [Ca^2+^]_i_ that is blocked by HC030031 or by siRNA-induced knockdown of TRPA1 [18]. The present study also confirmed it by using the conventional RT-PCR analysis, western blotting and immunocytochemical studies. The western blotting analysis of membrane protein also revealed a single band of TRPA1 protein, suggesting membrane expression of TRPA1 channel in hCFs. Similarly, Conklin et al. [8] showed TRPA1 expression in cardiomyocytes by the immunocytochemistry and western blotting analysis using the same antibody of TRPA1. TRPA1 responds to pungent plant compounds such as AITC and acrolein [33]. AITC is known as a selective and most potent agonist of TRPA1 channels [21, 22]. Activation of TRPA1 by AITC occurs through the covalent modification of cysteine residues located on intracellular amino (N)-terminus [34, 35]. And a lysine residue also contributes to AITC-induced activation of TRPA1 [34]. The present study showed the direct evidence for TRPA1 channel currents elicited by AITC and MG in hCFs. Replacement of extracellular Na^+^ to NMDG^+^markedly reduced the AITC-induced current. Furthermore, La^3+^ and RR, a general TRP channel blocker, inhibited the AITC-induced currents. The I–V relation of the AITC-induced current was almost linear with the reversal potential (Er) of approximately +0 mV. Outward rectification has been reported to be commonly observed when TRPA1 channels are activated by chemical agonists, intracellular Ca^2+^, and low temperature [1, 35, 36, 37]. However, it is also not uncommon that the linear I–V relationships are observed between −100 and +100 mV when the channel is strongly activated [38].

TRPA1 has been reported as a highly Ca-permeable nonselective cation channel [1, 39, 40] with an unitary conductance of 98 pS [22]. Recently, using the cell-attached patch techniques, Conklin *et al.* [8] showed single channel activity of the sarcolemmal patches enhanced by cinnamaldehyde, a known TRPA1 agonist. In the present study, AITC did not affect [Ca^2+^]_i_ in the absence of extracellular Ca^2+^, but in the presence of extracellular Ca^2+^, AITC increased [Ca^2+^]_**i**_. The AITC-induced [Ca^2+^]_i_ was blocked by RR or La^3+^ in a similar manner as AITC-induced nonselective cation currents. These results provide the direct evidence for functional existence of TRPA1 channels in hCFs, by using [Ca^2+^]_i_ measurements and the patch-clamp techniques. However, it remains unclarified whether basal activity of TRPA1 channels without agonists can contribute to basal [Ca^2+^]_i_. RR failed to affect basal [Ca^2+^]_i_ and background current, proposing that TRPA1 channels are less activated during basal conditions, compared with agonist stimulation. This is somewhat compatible with the following results. Andrei *et al.* [6, 7] reported that TRPA1 channel was present in cardiomyocytes and AITC increased the contractile function and [Ca^2+^]_i_ in cardiomyocytes, but not cardiomyocytes prepared from *Trpa1*^−/−^ mice. However, Bodkin *et al.* [40] showed that ventricular contraction under basal conditions did not change in *Trpa1*^−/−^ mice, suggesting that TRPA1 channels in basal state do not modify cardiac function. In the present study, La^3+^ further inhibited the background current with Er of approximately +0 mV, suggesting that hCFs have background nonselective cation channels other than TRPA1 as previously reported in various types of cells [30, 41, 42]. The further detailed studies including single channel recordings are required to identify the molecular basis of the background currents in hCFs.

Several lines of evidence have shown that Ca^2+^ entry plays an essential role in fibroblasts' biological functions [15, 43]. Previous studies have also reported that TRPA1 is associated with fibrosis in various tissues such as cardiac tissue [19, 44, 45]. Furthermore, cardiac fibroblasts not only produce ECM, but also mechanically and electrically bind to cardiomyocytes, affecting the electrical activity [46, 47]. Because TRPA1 is a nonselective cation channel, Na^+^ and Ca^2+^ pass the TRPA1 channel, resulting in membrane depolarization and action potential generation [48]. Thus, the activation of TRPA1 in hCFs may contribute to affect electrical activity in cardiomyocytes under the pathophysiological conditions. The TRPA1 channel has also been demonstrated to play an important role in mediating several pathophysiological conditions such as tissue injury and inflammation [2, 5, 9, 49]. Further, mounting evidence suggests that TRPA1 may be a key gatekeeper in regulating the inflammatory response [49] and oxidative stress [50], and play an important role in the pathophysiology of cardiac disease [9, 40]. Wang et al. [9] have reported that TRPA1 expression was increased in human failing heart. Conklin et al. [8] also showed that TRPA1 in cardiomyocytes is a target of lipid peroxidation-derived aldehydes, such as acrolein generated during ischemia-reperfusion, and TRPA1 activation may contribute to cardiac ischemia-reperfusion injury. Thus, TRPA1 appears to be involved in the regulation of cardiac function including fibroblasts under various conditions (myocardial infarction, heart failure, etc.) [5, 11]. The further research is required to clarify the pathological roles of TRPA1 in hCFs.

We should acknowledge several limitations. We had used the commercially cultured healthy hCFs in this experiment. Ion channels, receptor function and calcium regulation mechanisms in hCFs may be changed after the isolation and during the culture conditions. Furthermore, we used RR and La^3+^, a non-specific blocker, and the further studies using a more specific TRPA1 blocker and single channel recording techniques as well as freshly isolated hCFs are required to clarify the identification of TRPA1 channels in hCFs.

The present results provide the first evidence for existence of functional Ca^2+^-permeable nonselective cation currents activated by AITC and MG, possibly via TRPA1 in hCFs.

## Declarations

### Author contribution statement

T. Nakajima: Performed the experiments; Wrote the paper.

G. Oguri, H. Kikuchi and S. Obi: Conceived and designed the experiments; Performed the experiments.

F. Nakamura and I. Komuro: Contributed reagents, materials, analysis tools or data.

### Funding statement

S. Obi was supported by 10.13039/501100001691JSPS KAKENHI (18K08084) and T. Nakajima was supported by JSPS KAKENHI (19H03981).

### Data availability statement

Data included in article/supplementary material/referenced in article.

### Declaration of interests statement

The authors declare no conflict of interest.

### Additional information

No additional information is available for this paper.

## Figures and Tables

**SupplmentFig2A.TIF:**
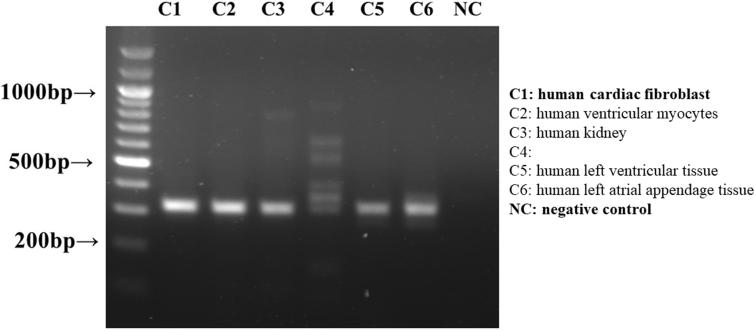


**SupplementFig2B.TIF:**
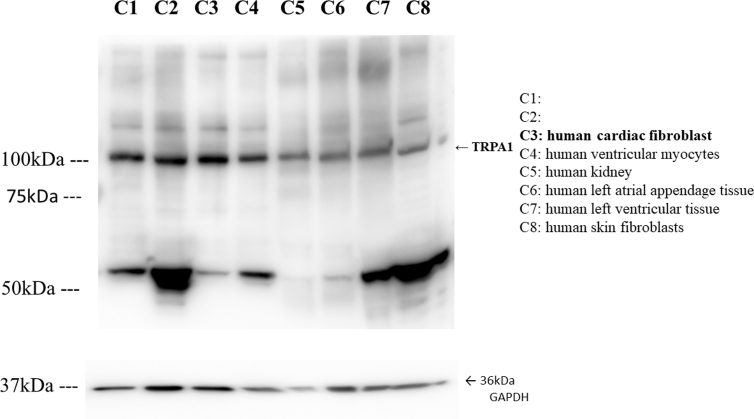


**SupplemntFig2C.TIF:**
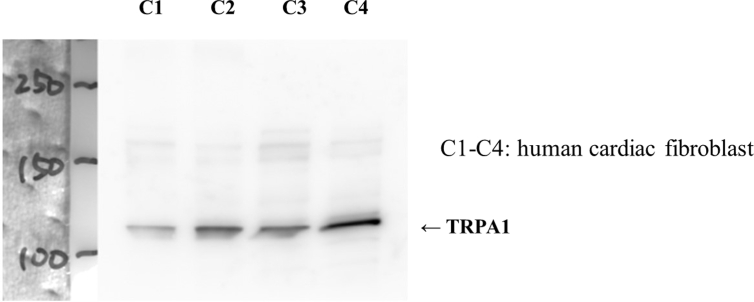


## Appendix A Supplementary data

Supplemental Figurelegends.docx Supplemental Figurelegends.docx

## References

[1] Story G.M., Peier A.M., Reeve A.J., Eid S.R., Mosbacher J., Hricik T.R. (2003). ANKTM1, a TRP-like channel expressed in nociceptive neurons, is activated by cold temperatures. Cell.

[2] Nilius B., Szallasi A. (2014). Transient receptor potential channels as drug targets: from the science of basic research to the art of medicine. Pharmacol. Rev..

[3] Mukhopadhyay I., Gomes P., Aranake S., Shetty M., Karnik P., Damle M. (2011). Expression of functional TRPA1 receptor on human lung fibroblast and epithelial cells. J. Recept. Signal Transduct. Res..

[4] Qian X., Francis M., Solodushko V., Earley S., Taylor M.S. (2013). Recruitment of dynamic endothelial Ca2+ signals by the TRPA1 channel activator AITC in rat cerebral arteries. Microcirculation.

[5] Wang Z., Ye D., Ye J., Wang M., Liu J., Jiang H. (2019). The TRPA1 channel in the cardiovascular system: promising features and challenges. Front. Pharmacol..

[6] Andrei S.R., Ghosh M., Sinharoy P., Dey S., IN Bratz, Damron D.S. (2017). TRPA1 ion channel stimulation enhances cardiomyocyte contractile function via a CaMKII-dependent pathway. Channels (Austin).

[7] Andrei S.R., Sinharoy P., Bratz I.N., Damron D.S. (2016). TRPA1 is functionally co-expressed with TRPV1 in cardiac muscle: Co-localization at z-discs, costameres and intercalated discs. Channels (Austin).

[8] Conklin D.J., Guo Y., Nystoriak M.A., Jagatheesan G., Obal D., Kilfoil P.J. (2019). TRPA1 channel contributes to myocardial ischemia-reperfusion injury. Am. J. Physiol. Heart Circ. Physiol..

[9] Wang Z., Wang M., Liu J., Ye J., Jiang H., Xu Y. (2018). Inhibition of TRPA1 attenuates doxorubicin-induced acute cardiotoxicity by suppressing oxidative stress, the inflammatory response, and endoplasmic reticulum stress. Oxid Med. Cell. Longev..

[10] Wang Z., Xu Y., Wang M., Ye J., Liu J., Jiang H. (2018). TRPA1 inhibition ameliorates pressure overload-induced cardiac hypertrophy and fibrosis in mice. EBioMedicine.

[11] Viana F. (2016). TRPA1 channels: molecular sentinels of cellular stress and tissue damage. J. Physiol..

[12] Zhang J., Li Y., Bai X., Li Y., Shi J., Hu D. (2018). Recent advances in hypertrophic scar. Histol. Histopathol..

[13] Schirone L., Forte M., Palmerio S., Yee D., Nocella C., Angelini F. (2017). A review of the molecular mechanisms underlying the development and progression of cardiac remodeling. Oxid Med.Cell.Longev..

[14] Moore-Morris T., Guimaraes-Camboa N., Yutzey K.E., Puceat M., Evans S.M. (2015). Cardiac fibroblasts: from development to heart failure. J.Mol.Med.(Berl)..

[15] Yue Z., Zhang Y., Xie J., Jiang J., Yue L. (2013). Transient receptor potential (TRP) channels and cardiac fibrosis. Curr. Top. Med. Chem..

[16] Minke B., Cook B. (2002). TRP channel proteins and signal transduction. Physiol. Rev..

[17] Hatano N., Itoh Y., Muraki K. (2009). Cardiac fibroblasts have functional TRPV4 activated by 4alpha-phorbol 12,13-didecanoate. Life Sci..

[18] Oguri G., Nakajima T., Yamamoto Y., Takano N., Tanaka T., Kikuchi H. (2014). Effects of methylglyoxal on human cardiac fibroblast: roles of transient receptor potential ankyrin 1 (TRPA1) channels. Am. J. Physiol. Heart Circ. Physiol..

[19] Okada Y., Shirai K., Reinach P.S., Kitano-Izutani A., Miyajima M., Flanders K.C. (2014). TRPA1 is required for TGF-beta signaling and its loss blocks inflammatory fibrosis in mouse corneal stroma. Lab. Invest..

[20] Ikeda K., Nakajima T., Yamamoto Y., Takano N., Tanaka T., Kikuchi H. (2013). Roles of transient receptor potential canonical (TRPC) channels and reverse-mode Na+/Ca2+ exchanger on cell proliferation in human cardiac fibroblasts: effects of transforming growth factor beta1. Cell Calcium.

[21] Capasso R., Aviello G., Romano B., Borrelli F., De Petrocellis L., Di Marzo V. (2012). Modulation of mouse gastrointestinal motility by allyl isothiocyanate, a constituent of cruciferous vegetables (Brassicaceae): evidence for TRPA1-independent effects. Br. J. Pharmacol..

[22] Jordt S.E., Bautista D.M., Chuang H.H., McKemy D.D., Zygmunt P.M., Hogestatt E.D. (2004). Mustard oils and cannabinoids excite sensory nerve fibres through the TRP channel ANKTM1. Nature.

[23] Bandell M., Story G.M., Hwang S.W., Viswanath V., Eid S.R., Petrus M.J. (2004). Noxious cold ion channel TRPA1 is activated by pungent compounds and bradykinin. Neuron.

[24] Nagata K., Duggan A., Kumar G., Garcia-Anoveros J. (2005). Nociceptor and hair cell transducer properties of TRPA1, a channel for pain and hearing. J. Neurosci..

[25] Hu H., Tian J., Zhu Y., Wang C., Xiao R., Herz J.M. (2010). Activation of TRPA1 channels by fenamate nonsteroidal anti-inflammatory drugs. Pflügers Archiv..

[26] McNamara C.R., Mandel-Brehm J., Bautista D.M., Siemens J., Deranian K.L., Zhao M. (2007). TRPA1 mediates formalin-induced pain. Proc. Natl. Acad. Sci. U. S. A.

[27] Grynkiewicz G., Poenie M., Tsien R.Y. (1985). A new generation of Ca2+ indicators with greatly improved fluorescence properties. J. Biol. Chem..

[28] Nakajima T., Okuda Y., Chisaki K., Shin W.S., Iwasawa K., Morita T. (2000). Bile acids increase intracellular Ca(2+) concentration and nitric oxide production in vascular endothelial cells. Br. J. Pharmacol..

[29] Hamill O.P., Marty A., Neher E., Sakmann B., Sigworth F.J. (1981). Improved patch-clamp techniques for high-resolution current recording from cells and cell-free membrane patches. Pflügers Archiv.

[30] Terasawa K., Nakajima T., Iida H., Iwasawa K., Oonuma H., Jo T. (2002). Nonselective cation currents regulate membrane potential of rabbit coronary arterial cell: modulation by lysophosphatidylcholine. Circulation.

[31] Clapham D.E., Julius D., Montell C., Schultz G. (2005). International Union of Pharmacology. XLIX. Nomenclature and structure-function relationships of transient receptor potential channels. Pharmacol. Rev..

[32] Jaquemar D., Schenker T., Trueb B. (1999). An ankyrin-like protein with transmembrane domains is specifically lost after oncogenic transformation of human fibroblasts. J. Biol. Chem..

[33] Bautista D.M., Jordt S.E., Nikai T., Tsuruda P.R., Read A.J., Poblete J. (2006). TRPA1 mediates the inflammatory actions of environmental irritants and proalgesic agents. Cell.

[34] Hinman A., Chuang H.H., Bautista D.M., Julius D. (2006). TRP channel activation by reversible covalent modification. Proc. Natl. Acad. Sci. U. S. A.

[35] Macpherson L.J., Dubin A.E., Evans M.J., Marr F., Schultz P.G., Cravatt B.F. (2007). Noxious compounds activate TRPA1 ion channels through covalent modification of cysteines. Nature.

[36] Zurborg S., Yurgionas B., Jira J.A., Caspani O., Heppenstall P.A. (2007). Direct activation of the ion channel TRPA1 by Ca2+. Nat. Neurosci..

[37] Wan X., Lu Y., Chen X., Xiong J., Zhou Y., Li P. (2014). Bimodal voltage dependence of TRPA1: mutations of a key pore helix residue reveal strong intrinsic voltage-dependent inactivation. Pflügers Archiv..

[38] Karashima Y., Prenen J., Talavera K., Janssens A., Voets T., Nilius B. (2010). Agonist-induced changes in Ca(2+) permeation through the nociceptor cation channel TRPA1. Biophys. J..

[39] Wang Y.Y., Chang R.B., Waters H.N., McKemy D.D., Liman E.R. (2008). The nociceptor ion channel TRPA1 is potentiated and inactivated by permeating calcium ions. J. Biol. Chem..

[40] Bodkin J.V., Thakore P., Aubdool A.A., Liang L., Fernandes E.S., Nandi M. (2014). Investigating the potential role of TRPA1 in locomotion and cardiovascular control during hypertension. Pharmacol.Res.Perspect..

[41] Bae Y.M., Park M.K., Lee S.H., Ho W.K., Earm Y.E. (1999). Contribution of Ca2+-activated K+ channels and non-selective cation channels to membrane potential of pulmonary arterial smooth muscle cells of the rabbit. J. Physiol..

[42] Mubagwa K., Stengl M., Flameng W. (1997). Extracellular divalent cations block a cation non-selective conductance unrelated to calcium channels in rat cardiac muscle. J. Physiol..

[43] Thodeti C.K., Paruchuri S., Meszaros J.G. (2013). A TRP to cardiac fibroblast differentiation. Channels (Austin).

[44] Yang Y.S., Cho S.I., Choi M.G., Choi Y.H., Kwak I.S., Park C.W. (2015). Increased expression of three types of transient receptor potential channels (TRPA1, TRPV4 and TRPV3) in burn scars with post-burn pruritus. Acta Derm. Venereol..

[45] Hirota S.A. (2018). TRPing up fibrosis: a novel role for TRPA1 in intestinal myofibroblasts. Cell. Mol. Gastroenterol. Hepatol..

[46] Kakkar R., Lee R.T. (2010). Intramyocardial fibroblast myocyte communication. Circ. Res..

[47] Abramochkin D.V., Lozinsky I.T., Kamkin A. (2014). Influence of mechanical stress on fibroblast-myocyte interactions in mammalian heart. J. Mol. Cell. Cardiol..

[48] Zygmunt P.M., Hogestatt E.D. (2014). Trpa1. Handb. Exp. Pharmacol..

[49] Bautista D.M., Jordt S.E., Nikai T., Tsuruda P.R., Read A.J., Poblete J. (2006). TRPA1 mediates the inflammatory actions of environmental irritants and proalgesic agents. Cell.

[50] Andersson D.A., Gentry C., Moss S., Bevan S. (2008). Transient receptor potential A1 is a sensory receptor for multiple products of oxidative stress. J. Neurosci..

