# Supplementary material for: Allyl isothiocyanate (AITC) activates nonselective cation currents in human cardiac fibroblasts: possible involvement of TRPA1
Source: Heliyon. 2020 Dec 31;7(1):e05816. doi: 10.1016/j.heliyon.2020.e05816 (PMC7797518; doi:10.1016/j.heliyon.2020.e05816)
Supplement: Supplemental Figurelegends.docx [file mmc1.docx]

**Supplemental Figures**

Figure 2A　RT-PCR analysis of TRPA1 in human cardiac fibroblasts and other cell types.

Figure 2B. Western blotting analysis of total cells for TRPA1 in human cardiac fibroblasts and other cell types.

Figure 2C: Western blotting analysis of membrane fraction for TRPA1 in human cardiac fibroblasts and other cell types.
